# Shared parenting and father involvement after divorce in Denmark

**DOI:** 10.3389/fpsyg.2023.1223574

**Published:** 2023-11-07

**Authors:** Kristian Sandberg

**Affiliations:** Center of Functionally Integrative Neuroscience, Aarhus University, Aarhus, Denmark

**Keywords:** shared parenting, joint physical custody, father involvement, divorce, mental health, well-being, dual residence, parent–child relationship

## Abstract

The Scandinavian countries make interesting samples for the study of shared parenting as they are characterized by some of the highest levels of father involvement and gender equality globally. Despite numerous studies, data from Denmark is noticeably absent in the international debate, partly due to a researcher preference for publishing in Danish. Here, I present an overview of the increase in father involvement in Denmark since the 1960s and on the increase in shared parenting across recent decades. I further examine Danish law, ministerial guidelines and guidelines from major Danish public and private institutions/organizations involved in deciding or advising on parenting practices post-divorce. I relate these to international research findings as well as to findings from Danish research. Overall, I find that Danish guidelines/practice have several reservations against shared parenting and substantial father involvement, which are not considered warranted by a substantial number of scientists and which are not supported by the majority of the available evidence. It thus appears that societal transition toward increased shared parenting has happened on a largely voluntary basis in spite of official law/practice. Updated law and/or ministerial guidelines are likely necessary if politicians desire that children experience the same high degree of father involvement post-divorce that they experience in society in general.

## 1. Background and definitions

The Scandinavian countries form interesting samples for the study of post-divorce family organization and shared parenting due to their high degree of gender equality and father involvement in childcare. Numerous studies are published internationally from particularly Sweden but also Norway. However, data from the third Scandinavian country, Denmark, is remarkably absent in the international literature. This is not because studies are not conducted, but rather that they have been published in Danish, and thus are not easily accessible to readers outside of Scandinavia. A main purpose of this article is to remedy this situation by conducting a detailed analysis of the development of shared parenting in Denmark over recent decades. Specifically, I review the literature on the development in custody and parenting time in Denmark in the context of local and international research on the topic, and I discuss this research in relation to Danish law, official guidelines and legal practice. The review examines whether the historical increase in shared parenting has happened on a voluntary basis or whether it has been facilitated by law/professional guidelines, and it raises the question of whether joint physical custody should be a legal presumption.

The scientific literature on the topic discusses parenting time using a set of terms that are to some extent bound to specific societies/laws. For example, the English-language literature often uses terms from the US legal system: joint custody (JC) and sole custody (SC). These can be further elaborated to specify if the custody is physical or legal: sole/joint physical custody (SPC/JPC) and sole/joint legal custody (SLC/JLC). JPC and SPC are defined on the basis of how much time the child spends with each parent. In some older studies, JPC is defined as children spending at least 25% of the time with each parent (i.e., having at least a 25–75 division of time with the parents) (Bauserman, 2002) whereas recently, it is more commonly defined as children spending at least 35% or even 50% of their time with each parent (for an overview of definitions in 40 studies between 2007 and 2018, see Steinbach, 2019). JLC refers to the legal right to be involved in major decisions about a child’s life and does not, as such, set any rule on how often the parent and child are physically together. Nevertheless, there is of course in practice a relationship so that a parent with legal custody on average spends more time with their child than one without. I use these terms primarily when discussing research in which clear definitions are made.

The terms and definitions in Danish law and practice are in many ways comparable to those in the US system, but also differs in some aspects. It is legally split into three separate domains: custody, residence and visitation. In Danish law (“Forældreansvarsloven”), custody refers to legal custody exclusively, and it is estimated to be shared in over 90% of the cases (Ottosen, 2016, p. 37). In the same law, residence refers to where the child is registered to live, and this is nearly always in one place (as I describe in Section 3). Since 2019, the law has technically allowed shared residence if both parents agree, but it can be argued that it has little to no legal significance for a number of reasons. For example, the law establishes that it can only be introduced voluntarily, it can be revoked unilaterally, it cannot be established in court, and even when it is in place, the child is still formally listed as residing in only one place for most purposes in public records. The residential parent has a number of rights above those of the non-residential parent, including, for example, the right to relocate with the child to anywhere within the country. Visitation is typically set (voluntarily, by mediation or by court) as a specific number of days across a 14-day period. For example, an equal division of 7 days with each parent is referred to as a 7–7 arrangement. In everyday conversation, typically only 7–7 is considered shared parenting. In Danish scientific studies, shared parenting is often referred to as an “equally split arrangement” (“lige deleordning”) or simply “split arrangement” (“deleordning”), and it includes typically only 8–6, 7–7 and similar divisions (e.g., Ottosen et al., 2018, p. 102; Ottosen and Stage, 2012, p. 14). Legally, the residential parent typically cannot claim child support from the other parent in an 8–6 or a 7–7 division. Comparing to the international literature where JPC is frequently used to describe 30–35% of the time with each parent, the Danish equivalent of JPC is thus defined relatively high as 43% (an 8–6 split) or more time with each parent. To avoid confusion based on differences in definitions, I generally avoid the term JPC when discussing Danish research/guidelines, and instead use the broader term shared parenting (which I also use when discussing the concept in general) or equal time/equal parenting time when a more specific definition is warranted.

In this article, I first provide an overview of the developments in parental caregiving time across recent decades for Danish men and women. Next, I compare men’s share of caregiving after divorce^1^ to that in society in general and establish that there is a substantial gap (with divorced men providing less care than men in society in general). Subsequently, I examine how Danish law and official guidelines might contribute to this gap. In order to examine whether reduced post-divorce father involvement could have a negative impact on children, I review both the international and Danish research literature on the topic in the context of Danish law and guidelines. The review focuses on the overall impact of parenting time, but also considers specific situations – for example when divorce involves young children or high interparental conflict. Finally, I report studies of children’s view on increased post-divorce father involvement, and I present researcher/expert consensus statements.

## 2. Changes in the division of labor

As in many other countries, the division of labor and parental roles in Denmark has changed dramatically over the past two generations, and equal divisions are closer than ever historically. I first examine this change and subsequently compare it to changes in children’s residence and to time spent with each parent post-divorce.

**Figure 1:**
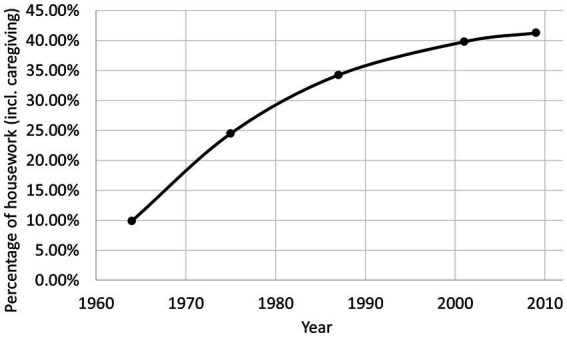
Danish Men’s share of housework in the period 1964–2009. Calculations are made using data on the number of hours spent on housework for men and women, respectively, as reported by Bonke (2012, Table 4.3) for the years 1964, 1975, 1987, 2001, and 2009. Men’s share of the housework is calculated as the number of hours spent by men divided by the total number of hours spent by men and women for each year. Parental caregiving is grouped as part of the housework in the report.

Over the past 60 years in Denmark, a dramatic change is evident both in terms of how time is spent overall and how women and men spend their time, respectively. Bonke (2012) presents an overview of this development, dividing time spent into work (paid labor), housework (a broad grouping of all unpaid work at home, including parental caregiving) and leisure time (including sleep). Using the data from Bonke’s (2012, Table 4.3) of the time spent in each of these categories by men and women between 1964 and 2009, a number of observations can be made and the development in men’s share of housework can be calculated. For example, Bonke (2012, Table 4.3) reports that in 1964, Danish men worked an average of 6 h per day (all year round) compared to 4 h in 2009, and the time gained has been transferred almost one to one to housework, which has increased from just under half an hour a day to 2 h and 17 min. This corresponds to an increase from 10 to 40% in men’s share of the housework (Figure 1). Interestingly, the additional 1 h and 45 min spent on housework by men has resulted in just approximately 45 min less housework and more professional work for women on average (Bonke, 2012, Table 4.3). A recent report with data from 2018 shows a continuation of the tendency with men performing 46% of the housework (Bonke and Wiese Christensen, 2018, Table 3.3). Overall, leisure time has increased slightly over the years, but so has the total amount of time spent on housework despite more household appliances. The question is how the extra time is spent?

The children seem to be a significant part of the answer. In 2008, fathers and mothers both spent approximately 30–40 min more each day on primary caregiving of children than fathers/mothers did in 1987 (Bonke, 2009, Table 5.2). In that period, the fathers’ share increased from 33 to 39%, and the share was relatively stable for children of different ages (Bonke, 2009, Figure 5.2).^2^ The fathers’ share of the total housework thus seems to correspond roughly to their share of caring for the children, *cf.* Figure 1. It may be noted that a large part of the average increase in caregiving time for fathers is due to far more fathers actively participating in childcare on a given day rather than them spending more time on their active days. Specifically, the probability of a father spending any caregiving time on a given day doubled from 31% in 1987 to 61% in 2009, but the time spent on an “active” day increased only from 1 h and 11 min to 1 h and 27 min. Perhaps surprisingly, the figures show that fathers spent more time with the children on average in 2001 and 2009 than mothers did in 1987. When counting also secondary caregiving (caregiving while performing another activity), fathers provided around 50% of the care when the difference in parent education level was 6 or fewer years and 40% for larger educational gaps already in 2001 (Bonke, 2009, Table 5.6).

A recent report shows that in society in general, 19% of men and 27% of women provide caregiving on a given day, and they spend 2 h and 54 min and 3 h and 16 min respectively, leading to an overall nearly identical contribution (47%) on active days but an overall contribution of 38% for men due to the fewer active days (Bonke and Wiese Christensen, 2018, Table 5.4). When examining parents exclusively, more caregiving time was spent on younger children, but fathers’ share of care was 38–39% for both young (under 7 years) and older children (Bonke and Wiese Christensen, 2018, Table 5.5).

The trend of increased time with the children for both mothers and fathers, as well as generally increased father involvement, can be found in other western countries, e.g., in a study with data from 13 western countries, incl. Denmark (Dotti Sani and Treas, 2016). Based on the study’s graphs, the fathers’ share of the care can be calculated to be approximately 35% in the period 2000–2010 in for example the United States, Canada, Norway, Netherlands, and the United Kingdom.

Overall, it can thus be said that both mothers and fathers spend significantly more time with their children than before, and despite the fact that Danish fathers in 2009 and 2016 were “only” responsible for approximately 40% of the primary care of children, they spent more time with them than mothers did 1–2 generations before that. It may further be noted that Figure 1 appears to have an asymptote below 45% and primary caregiving does not seem to exceed 40%, indicating that something is preventing a fully equal distribution of responsibilities, perhaps primarily that men appear to not reach the same number of active caregiving days as women.

## 3. Changes after divorce

Fathers’ increased total and relative time with the children is reflected to some extent in post-divorce custody in Denmark and internationally. An overview from Wisconsin, United States, shows for example, that SC has always been the most common outcome, but the proportion of children in such an arrangement decreased from around 80% in 1988 to around 40% in 2008 (Cancian et al., 2014). During that period, the proportion of children who lived primarily with their father remained more or less unchanged between 5 and 10%, while equal and unequal shared custody increased from around 5% each to around 25 and 20%, respectively. An even greater increase is observed in Sweden, where the proportion of children with shared residence and equal parenting time increased from around 1% to 30–40% (Bergström et al., 2015). Recent figures from other comparable countries show a similar development in JPC with an increase from 10% in 2002 to around 30% in 2012 in Norway (Kitterød and Wiik, 2017) and from around 10% in the early 1990s to 33% in 2006–2008 in Flanders, Belgium (Sodermans et al., 2013). Canada stands out with only 9% reported in a 2009 article (Swiss and Le Bourdais, 2009) and Australia also with only 8% (Cashmore et al., 2010).

Recent figures for residence and visitation in Denmark are reported, for example, in publications from The Danish Center for Social Science Research. Every four years (in 2010, 2014, 2018 and 2022), the center has published a report on the welfare and wellbeing of children – including children of divorce – in Denmark, and they have occasionally published research reports fully dedicated to shared parenting (in 2011 and 2012). One such publication reports a longitudinal study that followed children born in 1995. Here, Ottosen and Stage (2012, Figure 3.3) report that residence was almost always registered with the mother – in 100% of the cases for children aged 4–5 months, 92% at 7 years and 88% at 15 years. In a report from 2018, only a minor change in the asymmetry is seen: 88% at 7 years, 83% at 15 years (Ottosen et al., 2018, Figure 5.1.3). The 2022 report grouped children by whether they lived with their mother (53%) or father (6%), or whether they lived equally with both (41%) (Ottosen et al., 2022, Table 5.1.5). This means that for the children outside equal time arrangements, 90% (53%/(53% + 6%)) resided with their mother. They note that the probability of a child being in an equal time arrangement is largest if the residence is formally registered with their father, and that this probability increased gradually between 2009 and 2021 (Ottosen et al., 2022). This may be because many parents of two children, who practice shared parenting, register one child with each parent (Ottosen et al., 2022, p. 236). The small historic increase in paternal residence likely thus simply reflects an increase in shared parenting. Indeed, the proportion reporting living with the father (and not also with the mother) has been stable around 6–8% since 2009 (Ottosen et al., 2022, Table 5.1.5).

Ottosen and Stage (2012, Figure 3.4) further report that in this cohort, the proportion of children with equal parenting time arrangements varied by age, peaking at age 11 (around 18%), while it was rarer at ages 3 and 15 (both around 8%). Limited contact, weekend contact and extended contact (1–3, 4–6, and 7–11 monthly overnights, respectively) were consistently more prevalent at all ages. In general, with 15% in shared parenting schemes in 2009 (Ottosen et al., 2022, Table 5.1.3), Denmark thus ranked relatively low compared to many comparable countries despite general high societal father involvement. The proportion increased rapidly to 29% in 2013, 37% in 2017, and 41% in 2021 (Ottosen et al., 2022, Figure 5.1.3). This substantial increase across less than a decade is found for all age groups except for young children, where, for example, the prevalence for 3-year-olds decreased from 36% in 2013 to 21% in 2017 (Ottosen et al., 2018, Figure 5.1.4). The researchers behind the report speculate that this decrease could be related to official recommendations against shared parenting for young children or random fluctuation due to small samples in this category (Ottosen et al., 2018, p. 108). Based on the reports, it is possible to estimate the fathers’ share of care after divorce compared to society in general.

The 2012 report provides relatively precise definitions of the various visitation groups with the last measurement taken in 2011 when the children were 15 years old (and the earliest in 1996 when the children were 4–5 months). Knowing the probability of residence with the mother/father, respectively, for each age group, the proportion of children in each visitation group for each age group and how many nights the child spent with each parent, a fairly precise estimate can be made of how much time the children spent with their father and mother after a divorce.^3^ The figure is around 20–25% with their father at all ages. Therefore, it may be assumed that the day-to-day care after divorce around 10–20 years ago was provided primarily by the mother to an extent that differed significantly relative to what one would expect based on caregiving by fathers and mothers in society in general. After divorce, children spent 3–4 times as much time with their mother as with their father (an 75–80% versus 20–25% division), while mothers in general “only” provided 50% more primary care than fathers in society in general (a 60 to 40% division). In other words, the father’s share in care after divorce around 2000–2010 was less than the average father’s share in the overall housework in 1975 and thus approximately 30–40 years behind the development in society in general.

Corresponding calculations based on the 2014, 2018, and 2022 reports are somewhat less precise, as the visitation groups were defined more loosely and subjectively. In the latter reports, for example, it is divided into “no contact,” “shared parenting,” (meaning complete or almost completely equal time) and “other,” where the latter thus covers the three intermediate arrangements of the 2012 report. The 2022 report contains the numbers for all years so estimations can be made based on this report alone. If we assume an equal division of children reported as having the “other” arrangement into the three visitation groups from the 2012 report, the fathers’ share childcare post-divorce is estimated to just below 30% in 2013 and around 30–35% in 2017 for children of most ages. However, the younger children are again the exception, where the fathers’ share is down to 25% in 2017 after having been at nearly 30% in 2013. The assumption of an equal division appears relatively accurate as the more precise 2011 paternal caregiving estimates are generally −1.5% percent higher than the 2009 estimates and 3–8% lower than the 2013 estimates across the various age categories.

Using data from the 2022 report (Ottosen et al., 2022), fathers’ overall share of caregiving can be estimated to be around 33% in general in 2021. The share varies by age group from 25% for 3-year-olds to around 35% for 11- and 15-year-olds). Overall, fathers’ share in caring for the children after divorce in 2021 thus corresponded to the average father’s involvement in the period 1987–2001 [where it was 33–34% according to Bonke (2009)]. It was thus “only” 20–35 years after the general development in society despite a substantial increase in shared parenting. Specifically for young children aged 3, the involvement nevertheless corresponded to that of the 1970s.

In principle, there can be many reasons why fathers’ involvement in childcare after divorce lags decades behind general societal development such as the children’s/parents’ wishes, the children’s needs (and parents’ perception of this) and the practice of the family law system. Common to all perspectives appear to be a desire to act in the child’s best interests, but there are different perceptions of what that is. Below, I examine the current recommendations of Danish institutions and authorities in the field and subsequently relate these to recent scientific research and consensus.

## 4. Danish law and guidelines

With respect to residency and visitation, the Danish law (“Forældreansvarsloven”) is remarkably vague. §4 establishes that decisions must be based on the child’s best interests, without further specifying these except with respect to physical violence. §17 establishes that the courts have the authority to decide the child’s residence if the parents disagree. Importantly, §18a establishes that shared residency can only be established voluntarily (and thus not decided by the authorities), and it can be revoked by one parent (whether or not the other agrees). In terms of visitation, §19 establishes that the child (i.e., not the parent) has a right to visitation with the non-residential parent, and §21 establishes that the extent is defined to be set based on concrete assessment of the child’s situation without specifying how. §42 further establishes that the minister of social affairs can set rules/guidelines for these aspects, and this is indeed done (Social-, Bolig- og Ældreministeriet, 2023).

Much of these ministerial guidelines relate to procedures and to considerations for complex cases (e.g., cases involving violence, mental illness or substance abuse) while relatively little space is dedicated to specific guidelines for deciding residency and visitation in non-complex cases where the parents disagree. For residency, section 4.2 of the ministerial guidelines primarily establish that emphasis may be placed on the parent–child attachment, the parents’ personal characteristics, and on how the child will react to potentially moving as a consequence of the decision. Gatekeeping behavior (primarily obstruction of visitation according to section 2.2 of the guidelines) may also be considered as well as the risk of violence or witnessing violence. For visitation, Section 5 of the guidelines lists that the decision may be based on the age and development of the child, the child’s own opinion, their everyday life and activities, prior contact, interparental collaboration, the personal characteristics of the parents, the distance between homes, contact to siblings and other practical matters. It lists that any arrangement can be set, but for equal time it is usually required that it should not affect the child’s school or social life, and it is a decisive requirement that parents can collaborate to create continuity between the two homes and allow for flexibility with respect to the child’s need for contact. Specific guidelines are also set for children under the age of 3. It is, for example, mentioned that within the first 5 months of the child’s life, frequent but brief visitation of less than an hour may be set, and that these can be increased with age. At around 9–12 months, overnights can be initiated. Together, the law and ministerial guidelines primarily establish that unless both parents agree to shared residence, a single parent holds the residence and the courts can decide who. Apart from the decision of equal visitation time, no explicit rules guide the verdicts, but some factors are listed that may (or may not) be considered, leaving a lot of power of decision to the courts and The Agency of Family Law (“Familieretshuset”), which is described below.

The Agency of Family Law is the first and, in many cases, only institution that parents encounter during divorce. The agency – for example – handles divorce applications, provides mediation between parties, provides advice on custody/visitation arrangements, conducts interviews with children, and they can assign residence/visitation temporarily and refer a case to court. The majority of families set a visitation scheme without any official involvement, but for the substantial minority – 23-30% (Ottosen, 2016, p. 59) – who do not, the process begins at the Agency of Family Law. The agency is also often the first place where parents seek information about their choice of visitation scheme. They act according to the law and ministerial guidelines, but in light of the vague framework set by these, they also have their own published guidelines. These guidelines are central to understanding the workings of the legal system in Denmark as they are more explicit, form the basis of the initial mediation/decision and thus largely reflect the consensus within system.

The Agency of Family Law have recently updated their visitation guidelines (November 2022) (Familieretshuset, 2022a), but the previous document is still on the website (Familieretshuset, 2022b), and it is the one you are referred to if you access the website via Google’s search engine. In both documents, emphasis is placed on the child’s age, previous contact to parents, distance between parents’ residences and parents’ ability to cooperate, as well as their personal relationship. I review both these guides below. In March 2023, the guide document was updated with a new date, but I was unable to identify any other changes from the November 2022 guide.

The previous guide listed some very specific recommendations for visitation and residence. It was stated that young children need a primary caregiver with whom the child resides. Initially overnight stays with the other parents are discouraged, but contact may be gradually extended so that overnights can be attempted between the ages of 1 and 3 (i.e., somewhat later than mentioned in the ministerial guidelines). For children between ages 3 and 6, contact and the number of overnights can be increased, and if it works well for the child, shared time can be approached. For 6-12-year-olds, it is stated that nothing can be said about specific needs in relation to visitation schemes, apart from the fact that it can be important to listen to the child’s wishes. From the age of 12 and up, it is mentioned that children themselves typically do not desire shared parenting. Furthermore, it is mentioned that when there is a high level of conflict between parents, children should live primarily with one parent.

Overall, the previous guidelines focus on the relationship with the primary caregiver (typically the mother, considering the residency statistics), and then you may or may not gradually develop a relationship with the other parent, who would typically be the father, if circumstances allow for it. A very cautious attitude is expressed toward shared parenting both for young children, older children and in divorces with conflict or where parental cooperation is less than ideal. According to Ottosen and Stage (2012, Table 4.2), cooperation is less than “reasonable/tolerable” in 44% of all divorces in Denmark, and 59% of parents do not have “extensive cooperation.” If you take into account both age and cooperation/conflict, the recommendation was effectively that shared parenting at any given time is only suitable for a minority of children, around 15–20%, corresponding to the actual prevalence of shared parenting more than a decade earlier. It is also mentioned that not much is known about the effects of shared parenting, except that parental cooperation is crucial. In light of these recommendations, the decline in shared parenting of 3-year-olds since 2017 is unsurprising.

In the updated guidelines from November 2022 (Familieretshuset, 2022a), it is stated that research says that many children benefit from shared parenting if they are already closely attached to both parents, but that this does not mean it is always the right solution. In relation to specific ages, there has been a thorough rewrite, where focus is shifted from the importance of a primary caregiver and one home to a greater focus on relationships (plural). For young children aged 0 to 3 years, the guidelines say that the child can form attachments with multiple caregivers, and that both parents can have an important function if they are engaged in the daily care. However, it is still emphasized that young children need predictability and familiarity, and that this can be accommodated when the child resides with one parent and has frequent, short contact with the other. For 3-6-year-olds, it is specified that the number of successive overnights can be increased, while it is stated that 6-12-year-olds can be away from their (important) caregivers for a longer period of time. The guidelines mention that shared parenting is more common at this age, and that many children benefit from it. Older children are once again described as generally not wanting shared parenting.

In the recent guidelines (Familieretshuset, 2022a), research is summarized as indicating that there is insufficient knowledge about shared parenting of young children (0–3 years), but that it does not appear to be harmful to children aged 3–6 years, although it still is unclear which factors are decisive. Research findings are not mentioned specifically for older children. Parental cooperation is referred to as a generally important factor rather than something that affects different visitation schemes in different ways, and the statement that children should live primarily with one parent in cases of high interparental conflict has been removed. It is nevertheless emphasized that when children are asked, they report the parents’ cooperation as being of great importance to whether they thrive in shared parenting. A bibliography has also been included referring the reader to relevant research.

Overall, The Agency of Family Law appears to have changed their view from emphasizing the importance of a primary caregiver to the importance of caring relationships with both parents. The statements about young children’s need for unequal care have been replaced by statements that the research is unclear or does not indicate that children are harmed by shared parenting. Indeed, the recent guidelines indicate a greater openness to shared parenting overall (seemingly more open than the ministerial guidelines), but the specific examples of parenting plans are still based on young children having a single home and gradually seeing the other parent more until shared parenting can be approached around school age and likely abandoned again for teenagers. Statements about parental cooperation and low conflict are also toned down in relation to shared parenting and now appear to be viewed more as independently important factors, but there are still some cautious statements that might indicate reluctance toward shared parenting in case of conflict. Despite the less negative or reluctant attitude toward shared parenting in general, however, it is not presented as a general recommendation for most families, and research is only mentioned once as positively supporting it, followed by a sentence urging not to generalize.

In addition to state entities, at least two other major Danish organisations with significant funding provide support and advice in relation to divorce: Mødrehjælpen (meaning “Mothers’ help”) and Børns Vilkår (meaning “Children’s conditions”). These express similar opinions. For example, Børns Vilkår writes that “it is your cooperation, level of conflict and responsiveness to the child that are most important for your child’s well-being – not where your child lives and sleeps,” and it is emphasized, that shared parenting requires extensive cooperation. Nevertheless, they do mention shared parenting in relation to relatively young children, and they mention in one example that it is something a four-year-old might suddenly need (Børns Vilkår, 2023). Interestingly, for teenagers, they mention that instead of moving between homes every week, children might need shifts 1–2 times a month (in contrast to seeing the non-residential parent less). In this way, Børns Vilkår’s recommendations seem clearly more open to shared parenting than The Agency of Family Law’s previous guide, while they still seem to place more emphasis on cooperation as a prerequisite than the new guide. Mødrehjælpen’s recommendations seem to be completely in line with the old guide and write that shared parenting places great demands on parents and children and, among other things, requires good cooperation (Mødrehjælpen, 2023).

Taken together, Danish law and ministerial guidelines are relatively vague and mostly provide a list of aspects that may be taken into account. Critically, however, the law establishes that when parents disagree, residency can be listed with one parent only, effectively establishing this parent as the primary caregiver. This parent has additional rights, and the child has preferential access to them as they must live at least half of the time with this parent. This means that shared parenting can only be practiced through the rules on visitation in case of disagreement. In that context, the ministerial guidelines place a hard requirement of interparental collaboration on equal time, meaning that this becomes difficult to establish outside of a mutual decision by the parents. Other organizations advising and taking part in the initial decisions on residence/visitation are relatively conservative and generally refrain from endorsing shared parenting as a default solution. Below, I review the latest research and relate it to Danish law/guidelines. The main focus is placed on The Agency of Family Law’s guides as these are both the most detailed/explicit in terms of recommendations and as the latest guide provides a list of specific references.

## 5. The impact of custody on children’s wellbeing

Studies of JC have measured a number of parameters both in relation to the child’s general wellbeing, mental health and academic ability immediately after divorce and later in life. An early meta-analysis (which appears on the literature list in The Agency of Family Law’s guide) showed that frequency of contact was generally not related to child outcome (Amato and Gilbreth, 1999), and this finding is still often referred to in both Danish and international literature, e.g., in Ottosen and Stage’s (2012, p. 78) analysis. The findings have since been replicated and extended, and often quality of contact (e.g., the father-child relationship and involvement in care activities) is emphasized over frequency (Adamsons and Johnson, 2013). However, it has been pointed out that the *frequency* of contact is not a meaningful measure of the *amount* of contact, and that the “quality” measures in reality reflect quantity (Fabricius, 2020). For example, a child with only two weekly hours of contact is scored as having a contact frequency of 4 times per month, while a child living with each parent in alternating weeks is scored at 2 times per month. The quality variables, in contrast, include how often the non-residential parent puts the child to bed or does homework with them, and of course this happens more often with more overnights. It has also been pointed out that time is a prerequisite for building and maintaining a close relationship so it is difficult to have quality without quantity (Adamsons, 2018).

When the amount of contact has been examined directly, the results are quite different. Children in JC typically do substantially better than children in SC as evidenced in two meta-analyses (neither of which are referenced by The Agency of Family Law). In an early meta-analysis of 33 studies, Bauserman (2002) compared JC and SC and found that children in JC did better than children in SC (and moreover not significantly different from children in intact families) on a wide range of parameters: general adjustment, family relationships, self-esteem, emotional and behavioral adjustment, and divorce-specific adjustment. In a subsequent meta-analysis, he also found that JC was associated with a better father-child relationship, less parenting stress, less interparental conflict and a lower relitigation rate, and better overall adjustment (Bauserman, 2012).

Most studies contrast SC and JC as dichotomous categories, but graded increases toward equal time have also been examined. A meta-analysis of 16 studies found a large number of benefits related to JPC, and the effects were greater for children who spent at least 40% of their time in each home compared to those who spent only 30–39% in one of the homes (Baude et al., 2016). Similarly, a Swedish study of around 148,000 children (including around 46,000 children from divorced families) found that psychosomatic symptoms decreased gradually as a function of parenting time (Bergström et al., 2015). Neither of these are referenced by The Agency of Family Law.

In Denmark, Ottosen et al. (2022, Table 5.2.6) report that the proportion of children that have a confidential relationship with their parents vary with respect to residence. A confidential relationship was defined as whether the child reported that it is “easy” or “very easy” for them to talk to the parent about a topic that really bothered the child. Children living with their father typically had a lower chance of having a confidential relationship with their mother than in intact or shared residence families, and similarly for the father-child relationship in maternal residence arrangements. The total amount of confidential relationships is very high for shared residence and similar to the numbers for intact families. While shared residence may be associated with a slight decrease in the probability of a confidential relationship with one parent compared to sole residence, this is compensated by a much larger increase in the probability of a confidential relationship with the other parent, thus increasing the total number of confidential relationships. Similar effects were found when examining whether children feel that their parents care for them (Ottosen et al., 2022, Table 5.2.8). Here, the numbers were nearly identical for shared parenting and intact families, but substantial drops were observed for the non-residential parent in sole residence families without any increase for the residential parent. Children in shared parenting arrangements thus had a high probability (around 85–94%) of having a caring relationship with each of their parents while children in sole residence arrangements at best had a similarly high probability for one parent (69–95%) but a much lower probability for the other parent (47–83%).

The literature thus consistently finds a positive relationship between equal parenting time and well-being of the children on a wide range of parameters, and equal parenting time is related to optimal parent–child relationships mirroring those found in intact families. Given the consistency of the findings, subsequent skepticism has focused on whether JC has a causal effect or whether the effect is due to other factors such as wealthy, educated, resourceful parents with low mutual conflict and older children self-selecting JC. The research has therefore tried to separate these factors in increasingly sophisticated designs to examine if equal time in itself has causal, positive effects. In the sections below, I review the literature on the proposed confounding factors.

## 6. The effect of interparental conflict

Interparental conflict in the context of divorce is particularly interesting as it has not only been proposed as a confounding factor (the claim that lower conflict is the cause of benefits of JPC), but also as one that interacts with the type of custody (the claim that for high-conflict couples, SC is best for the children). It is specifically mentioned in all the guidelines presented above, but at the same time, the level of conflict post-divorce frequently changes, and conflict is rarely ongoing for years. For example, the level of conflict is relatively high up to and immediately following divorce – when custody/residence is determined – but it declines afterwards (Fabricius and Braver, 2006). In Ottosen and Stage’s Danish sample (N = 919) from the 2012 analysis, none of the custodial parents who reported conflict in 2007 also reported it in 2011, while other custodial parents who did not previously report conflict now did (Ottosen and Stage, 2012, Table 4.4). In general, the proportion reporting conflict was very low already in 2007 (around 4%), which is presumably related to the fact that most divorces had occurred years in advance. The level was 4–5% for all visitation categories (apart from “no visitation,” where there was typically no contact between the parents and therefore no possibility of conflict).

**Figure 2:**
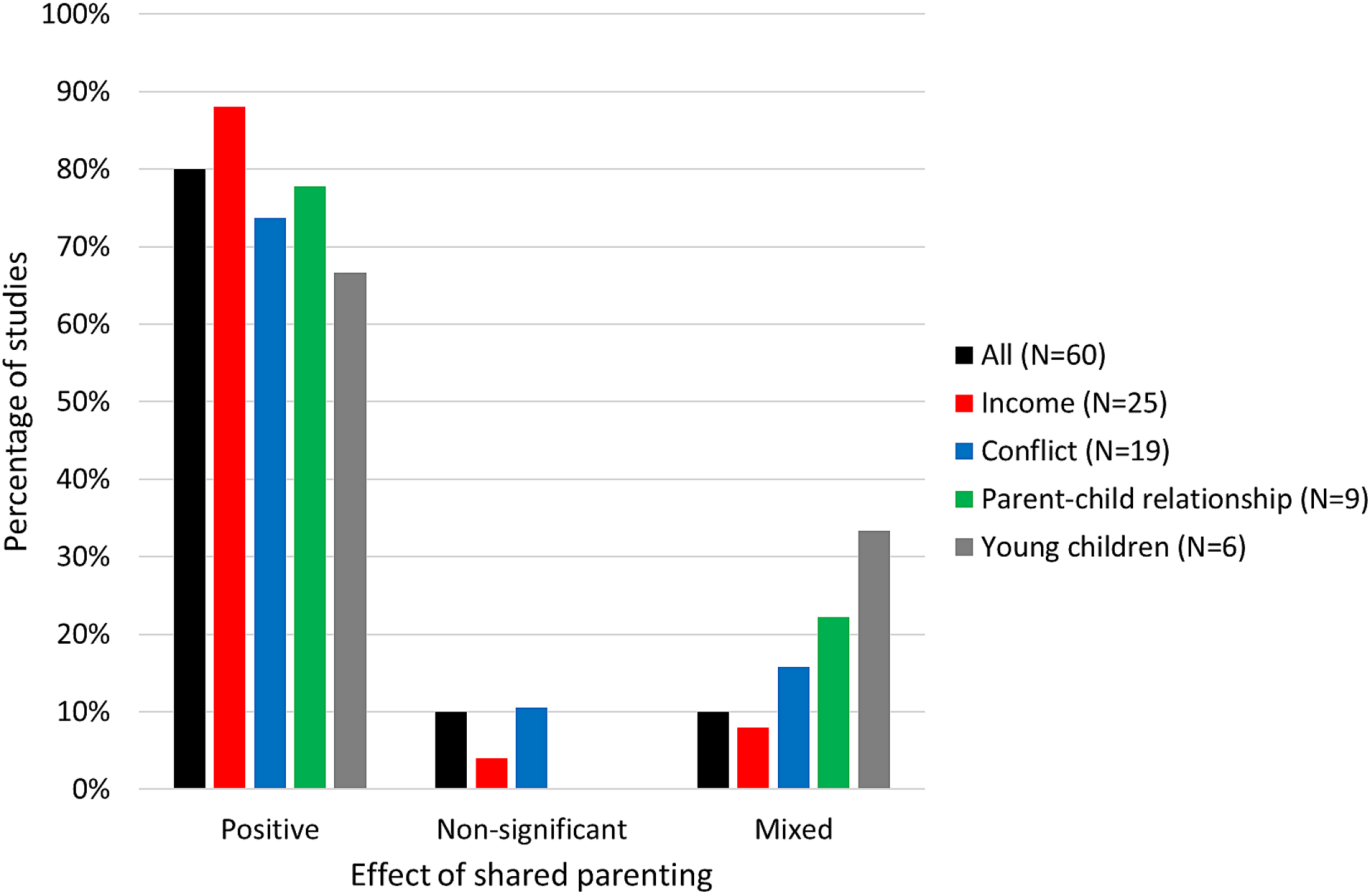
Studies of children’s outcome in JPC. Percentage of studies which – according to Nielsen (2018, 2021) – report positive (for some or all outcome variables), non-significant or mixed effects of JPC (no studies reported exclusively negative effects). The studies are shown jointly (first bar) and individually grouped according to whether they take into account parents’ income, interparental conflict, parent–child relationship and whether they studied young children (ages 0–5) exclusively.

Interestingly, Bauserman investigated conflict already in 2002 and did not find that it moderated the positive effects of JC, but he also noted that the data at the time was sparse (Bauserman, 2002). A more complete investigation was carried out by Nielsen in a review of 60 quantitative studies on JPC (Nielsen, 2018). She categorized studies according to the outcome and according to the additional factors (e.g., conflict) that the studies took into account. Based on the numbers reported by Nielsen, Figure 2 plots the percentage of studies showing positive, neutral (non-significant) and mixed outcomes (no study is reported to show exclusively negative outcomes). The figure is supplemented with information from Nielsen (2021) to include calculations for young children. Figure 2 shows that a clear majority of studies report increased well-being in JPC, both in general (45 of 60 studies) and when conflict was taken into account (14 of 19 studies). Nielsen dedicated three pages to a detailed discussion of the evidence and concluded that there is very little support for the view that reduced conflict explains the benefits of JPC.

Mahrer et al. (2018) conducted a detailed review of 11 studies on conflict. They found that conflict within 2–3 years of the time of divorce – i.e., when custody/residence is first determined – was not related to poorer outcomes for children in JPC. They mentioned that studies that controlled statistically for the level of conflict typically still found better outcomes for JPC (and for more versus less time with the father in SC in the studies that compared little or no contact to 25% or more time with the father). They also examined the effect of quality of care and concluded, for example, that high-quality parenting by at least one parent protects against negative effects of conflict. In terms of policy and practice, they concluded that there is no consistent set of findings that support a policy against shared parenting based on having a conflictual relationship at the time of divorce, and they argued that other factors (such as quality of parenting) should be weighed more heavily. Finally, it is worth mentioning that primarily older studies found negative effects of conflict, and that several of these studies did not examine JPC, but instead increased father contact in SC. This highlights the possibility that conflict is primarily harmful when combined with unequal parenting time compared to when there is no contact at all (which is in itself associated with poorer outcomes) as well as to when parenting time is equal.

The Agency of Family Law does not refer to the reviews of Nielsen or Mahrer and colleagues, but instead to two others by Steinbach (2019) and Berman and Daneback (2022). Compared to Bauserman, Nielsen and Mahrer, they adopt a somewhat different perspective.

Berman and Daneback (2022) argue that there is overall consensus on the benefits of shared parenting. Yet, the consensus only applies when there is no interparental conflict, when parents are able to cooperate and when the children are above 4 years old, thus effectively dividing scientists into two camps that they label advocates and opponents of shared parenting. They dedicate one paragraph to the topic of conflict, and they reference only a subset of the available articles. They mention that conflict increases behavioral and psychosocial problems (and thus is a general negative factor), and they highlight, with reference to five studies, that dual residence can be a bad solution for some children as they are exposed to conflict. They reference only one of the 14 studies identified by Nielsen showing benefits of JPC when taking into account conflict. Nevertheless, they subsequently mention, with reference to – for example – Nielsen’s and Mahrer’s reviews that others argue that the negative effects of conflict are more than outweighed by the positive effects of having a relationship with both parents, and that conflict might only be harmful if it is persistent. In the discussion section, they conclude conservatively that children benefit from dual residence when conflict is low. This conclusion seems somewhat in contrast to earlier parts of their review where they emphasized that there is no consensus on this aspect, and that the debate is still ongoing.

Steinbach (2019) similarly mentions that there is currently consensus on the benefits of JPC when parents cooperate and have low levels of conflict. In her review of the studies generally showing benefits of JPC, Steinbach often focuses on potential confounds and mentions, for example, that the benefits in one study (Jablonska and Lindberg, 2007) became non-significant when the number of close friends and school satisfaction were controlled for. However, these appear to be very conservative control variables as one could easily imagine that if JPC has a causal effect on general well-being, psychological problems, physical health and cognitive development, then SC could result in a range of academic and social difficulties. If this is the case, controlling for them is in effect controlling for an outcome measure. In connection with the literature on conflict, Steinbach dedicates one paragraph (on p. 357) to theoretical arguments as well one (on p. 360) to empirical studies. She cites one study with a positive effect (Spruijt and Duindam, 2009) as well as two quantitative studies and one qualitative with mixed findings (McIntosh, 2009; Cashmore et al., 2010; Vanassche et al., 2013). Of the 14 studies reported by Nielsen (2018) as showing positive effects, only one (Spruijt and Duindam, 2009) is thus included in Steinbach’s review, and Steinbach refers to the studies labeled “mixed findings” by Nielsen as having identified negative findings.

Taken together, the reviews of Steinbach (2019) and Berman and Daneback (2022) dedicate relatively little space to the conflict literature whereas Nielsen (2018) and Mahrer et al. (2018) dedicate substantially more. While the quantity of space is not synonymous with the quality of a review, it does allow for a more detailed discussion and mention of all studies identified for the review. This might make it easier for a reader to judge the relative strength of evidence for each position themselves. The conclusions of the four reviews also differ quite substantially. Nielsen and Mahrer and colleagues argue that the level of conflict cannot explain the benefits of JPC and that the presence of conflict should not prevent JPC. In contrast, Steinbach and Berman and Danebach report that there is no consensus. The Agency of Family Law thus refers to the two literature reviews that convey the least positive view of JPC in case of conflict while they do not mention the two reviews that take a more positive view on JPC.

## 7. The effect of income, education, and parent–child relationship

Other factors such as parental income and the existing parent–child relationship have also been investigated. As can be seen in Figure 2; Nielsen (2018) found that these two factors could not explain the benefits of JPC. Furthermore, a number of Swedish studies controlled for education and other variables. For example, Bergström et al. (2018) found positive effects of JPC after controlling for parents’ level of education and country of birth. Fransson et al. (2018) found that the living conditions (with respect to economy, social relations, health, culture/leisure time) of shared parenting children were better than those of single-custodial-parent children even when controlling for the child’s sex and age as well as the parents’ education and country of birth. Bergström et al. (2015) found that the benefits of JPC remained when statistically controlling for parents’ age and country of origin as well as perceived wealth and (current) parent–child relationship. The latter is quite a conservative control variable as the relationship is likely related to the time spent together. In the Danish report by Ottosen et al. (2018), there was a positive effect of JPC, but it disappeared in models controlling the interparental relationship and parent–child relationships. They mention, however, in this connection precisely that one cannot conclude that time with each parent is irrelevant because relationships require time, and because their analysis showed that the relationship with the father and the mother had separate positive contributions to well-being.

## 8. The role of the Age of the children

Another topic mentioned in all of the above Danish recommendations is the age of the children. Here, there have been theoretical reasons as well as early research indicating a lack of benefits of JPC. However, recent studies are generally more positive. In a study of 3,656 children aged 3–5 years (including 287 children of divorce), Bergström et al. (2018) found that children in JPC had fewer psychological problems than children who lived primarily or exclusively with one parent – even when controlling for parents’ level of education and country of origin. Nielsen (2021) reported and discussed the results of six studies specifically examining young children (see Figure 2). She referred to two of the studies as controversial and pointed out that they were criticized in a consensus statement (Warshak, 2014) from 110 researchers and practitioners. One study (McIntosh et al., 2010) was criticized for using non-standardized tests, questionable interpretations of results, small samples of non-representative couples who had never lived together, and the study failed to mention positive effects. The second study (Tornello et al., 2013) has also been criticized for using non-standardized tests in a non-representative sample of minority parents living in impoverished areas with high rates of violence, abuse and mental health problems. In this study, too, the negative findings were emphasized, while the positive and non-significant findings were ignored or downplayed. The remaining four studies (Solomon, 1998; Pruett et al., 2004; Fabricius and Suh, 2017; Bergström et al., 2018) concluded that babies, toddlers and preschoolers who often spent the night with their father (up to equal time) did better overall than children who primarily spent their nights with their mother.

One of the very recent studies mentioned by Nielsen (2021) provides some interesting insights. Fabricius and Suh (2017) investigated the relationship between young adult children of divorce and their parents in relation to the degree of contact they had between ages 0–2 years. They observed positive effects on the young adult’s relationship to both parents as a function of overnight stays with the father in early childhood up to and including equal time. In other words, the best overall young adult-parent relationship was observed for the participants who – before the age of three – had a similar number of overnights with both their parents. The effect was found for overnights when the child was under 1 year old, but to an even greater extent for 2-year-olds. The results held after controlling for subsequent parent–child time as children/adolescents, parents’ level of education and conflict up to 5 years after the divorce. The father-child relationship improved gradually up to equal time. In contrast, the mother–child relationship improved primarily between 0 and 1–2 overnights with the father across a 14-day period and subsequently remained stable between 1–2 and 6–7 overnights. It thus appears that early equal contact is related to a better lasting father-child relationship without the mother–child relationship suffering from it. One of the measures that was used in the study (“mattering” – i.e., whether the child fells that it matters to the parent) has subsequently been found to be related to children’s mental health (Vélez et al., 2020).

Fransson et al. (2018) have published a short overview article of recent Swedish studies on the topic. They included three epidemiological studies and one interview study in their overview for young children. Based on the epidemiological studies, they concluded, for example, that young JPC children had fewer psychological and behavioral problems than young SPC children. In the interview study, they found that 24% of the interviewed parents did not initially agree to JPC and some of these did not trust the other parent’s ability to take care of the child. Nevertheless, the majority ended up being satisfied with JPC and feeling that their children benefitted from it. They focused on the positive effects of involved fathers as part of the explanation for the good results.

After this overview article, another Swedish study was published. Bergström et al. (2021) examined 12,845 3-year-old children, including 642 children of divorce, in relation to the connection between psychological well-being, JPC and parental cooperation. They found that 3-year-olds in JPC generally had fewer psychological problems, even when controlling for the parental level of education. After statistically controlling for parental cooperation, the findings were rather surprising, in that there were no significant differences between children in the different divorce categories, but children in intact families fared significantly worse than JPC children. This indicates that controlling for the level of cooperation may be too conservative as few would argue that parents should generally divorce for the sake of their children. A follow-up analysis was more informative and showed that good cooperation generally correlated with better mental health, but that the benefit was greatest in intact families and JPC. In other words, psychological well-being was roughly equally bad regardless of whether parental cooperation was good or bad when children lived exclusively or mostly with one parent, while children benefited from positive parental cooperation in JPC and intact families. While Bergström and colleagues do not mention it explicitly, it could thus be speculated that JPC might be a prerequisite for reaping the benefits of good parental cooperation in relation to psychological problems. It should be emphasized that SPC or inequal parental care constellations were not found to provide better well-being in the case of poor cooperation. There was thus no support for SPC or inequal care being a better choice in the absence of good collaboration as mentioned in the previous guide from The Agency of Family Law.

In her review article, Steinbach (2019) summarizes the results of only two studies on young children (Mcintosh et al., 2013; Tornello et al., 2013) despite the Swedish studies being mentioned elsewhere in the article. The two studies are cited as providing evidence against JPC, although it is acknowledged that the conclusions are debated. The position of the advocates of JPC is described as based on theoretical arguments from attachment theory, and it is accompanied by a remark that not only emotional support but also competency is required to care for a very small child. In contrast, Berman and Daneback (2022) highlight broader literature reviews and conclude that overnights with both parents are unproblematic, but more research is needed. Rather remarkably, they reference one study of Bergström et al. (2018) elsewhere, but do not mention it in relation to the findings on young children. In their discussion section, they are once again more conservative and write that research is too scarce to draw any conclusions for children under the age of four.

Of the four reviews presented above, The Agency of Family Law refers only to the latter two, of which at least one is very limited and both take a relatively conservative perspective on JPC and neglect to mention individual studies with positive outcomes. The Agency of Family Law does, however, additionally list one study by Bergström et al. (2018), but not the 2021 study (Bergström et al., 2021). The ministerial guidelines similarly appear more in line with the perspectives taken in the reviews with the most skeptical views of JPC.

## 9. Causality

Establishing the causal effects of different visitation arrangements is notoriously difficult as random, controlled trials obviously cannot be done. Instead, researchers have used a range of different methodologies to make inferences about causal effects. For example, parental relocation often causes abrupt, drastic changes in the amount of contact with one parent, and one can therefore examine the effect of moving on parent–child relationships. Braver et al. (2003) investigated this and found negative effects for children where one parent had relocated. These children experienced greater inner turmoil during the divorce/experienced it as more unpleasant, and they experienced less support from the noncustodial parent (regardless of which parent had moved and regardless of whether they themselves had moved). They further experienced to a lesser extent having two good role models. In a follow-up control analysis, it was ensured that the effects were not due to existing conflict/violence before moving (Fabricius and Braver, 2006).

Self-selection is typically considered the alternative to a causal explanation so another line of research has examined the extent of self-selection and attempted to rule this out as an explanatory factor. For example, it can be examined whether the benefits of shared parenting disappear when the parents initially oppose it, i.e., whether they have self-selected or (possibly reluctantly or after a court decision) have accepted it. Nielsen (2017) identified four studies (from the 1980s and 1990s) where a large proportion of the JPC families (between 40 and 82%) were initially in conflict regarding the custody arrangement. JPC children in these studies still fared better than SPC children, indicating that self-selection into JPC could not explain the benefits. In addition to these, Fabricius and Suh’s (2017) above-mentioned findings also held when there had been no agreement about shared parenting.

## 10. The children’s perspective

The Danish-language research literature places a prominent focus on the perspective of the children, often in qualitative studies, and this is reflected in the guides of The Agency of Family Law. A large, qualitative study of 200+ pages examines the experiences of children in shared parenting arrangements through interviews with 28 non-randomly selected children along with 24 parents and 4 adult children (Ottosen et al., 2011). As in the international literature on the topic, the study provides diverse and nuanced reports from the children of the perceived advantages and disadvantages of equal time with both parents, but it is difficult to generalize broadly. It is worth noting, however, that most children reported equal time to be a positive thing – whether or not it was established voluntarily (Ottosen et al., 2011, pp. 135–6). To obtain a more representative overview, it can be valuable to look at larger, quantitative studies. Fabricius and Hall (2000) investigated children’s perspectives in a larger sample of around 800 young adults whose parents had divorced during the young adults’ childhood. They asked what the participants themselves had preferred, what their parents had preferred, how it actually was, and what they and their parents generally thought was best for children. There was a general reported agreement between the wishes of the participants and their fathers, while the actual time with each parent corresponded to the perceived wishes of the mothers. Similarly, in the perception of what is generally best for children, the majority of the participants – in agreement with their fathers – reported that equal time with both parents is best, while they reported that the mothers thought less time with the father is better. In fact, 93% of participants who had experienced shared parenting reported that this is best for children, while children who had not had equal time with both parents reported that it is best for children to have either equal time or significant time with father (corresponding to ratings of 4 and 3, respectively, on a scale from 0 to 4).

**Figure 3:**
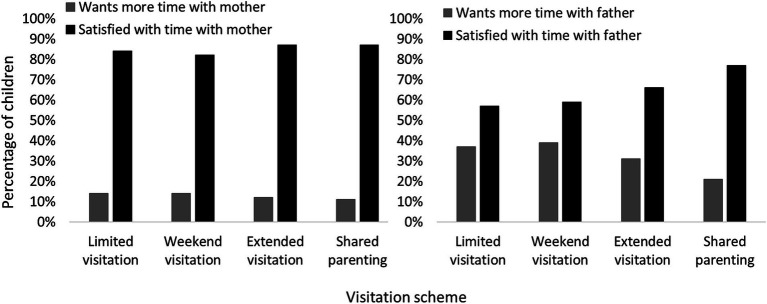
Danish children’s satisfaction with visitation. Reports of 11-year-old children of whether they desire more or have adequate time with their mother (left) and father (right) as a function of visitation scheme. Data from Ottosen and Stage (2012, Table 4.6).

A major quantitative Danish study has also been conducted on the topic. Ottosen and Stage (2012, Table 4.6) investigated 1,354 children’s wishes for more time with their father/mother across different types of visitation arrangements for children when they were 11 and 15 years old. Children were categorized as belonging to one of the following groups: No visitation (no visitation at all), limited visitation (has visitation but less than 3 nights per month), weekend visitation (visitation up to 6 nights per month), extended visitation (visitation up to 11 nights per month), and shared parenting (has approximately equal time with each parent; typically moving between homes every 7 or 14 days). The figures for the 11-year-olds are plotted in Figure 3 for all categories with visitation. It is evident that there were many more children who desired more time with their father compared to what they had, than there were children who desired more time with their mother compared to what they had. This unmet desire decreased as a function of overnights. Even for shared parenting, however, there were twice as many children who wanted more time with their father as there were children who wanted more time with their mother. Considering that around 90% of the children resided with the mother, this difference likely reflects that there was a significant minority of children who would have preferred living mostly with their father, but instead got equal time with both parents. The distribution for the 15-year-olds were in every way similar to those for the 11-year-olds, except that virtually everyone with shared parenting (about 95%) was satisfied with the time with the father as well as the mother.

Ottosen and Stage (2012) concluded that satisfaction generally increased with overnights (without commenting on the differences for fathers and mothers) and that overall satisfaction was greater for the 15-year-olds. It is worth taking a closer look at the second conclusion. Satisfaction for 15-year-olds compared to 11-year-olds was indeed higher for each visitation category considered separately without taking into account how many children were in each category: For example, the proportion of children who wanted to see their father more was reduced by 4, 11, 7 and 17% for each of the four categories leading to apparent substantially less dissatisfaction. Such a conclusion is, however, problematic as a large number of children (around 200) between ages 11 and 15 were moved to the “limited visitation” category (in accordance with the guidelines that teenagers do not want shared parenting), which had the greatest degree of dissatisfaction. Using the numbers provided by Ottosen and Stage (2012), the total proportion of children who wanted to see father more – independently of visitation category – can be calculated to be 33% for the 11-year-olds and 30% for the 15-year-olds. The overall proportion of dissatisfied children was thus relatively similar at 11 and 15 years. One interpretation is that the desires of the children who wanted less time with their father were met as they got older, while those who wanted a more equal arrangement still did not have their wish fulfilled and risked even less time with him, resulting in highly similar levels of dissatisfaction in combination with less time with the father on average.

Overall, it thus appears that, both in Denmark and internationally, children’s desires are taken significantly more into account when they want time with their mother than when they want time with their father, and at the same time, it is the children’s impression that the mother has significantly more power in relation to determining custody/visitation than they themselves and their father have had. The issue is evident in Danish research, but it is not reported outside a table listing. Presumably for this reason, The Agency of Family Law does not refer to it, and it is not mentioned in ministerial guidelines.

## 11. Consensus reports and expert evaluations

Several international groups of experts have published consensus reports or conclusions from panel discussions, but these do not appear on The Agency of Family Law’s reference list. In the most recent consensus statement (Warshak, 2014) from 110 researchers and practitioners, most Nordic countries were represented (Sweden was represented by five experts, for example), but Denmark was noticeably absent. The report concluded the following: 1) Shared parenting (typically defined in the literature as at least 35% of the time with each parent) should be the norm for children of all ages, incl. very small children. 2) Children under the age of 4 should have the opportunity for overnight stays with both parents. The alternative of only spending a few hours together several times a week stresses the parent–child relationship. There is no evidence that infants and young children should not have frequent contact, including overnights with both parents. 3) The recommendations apply generally to most parents/children. The exceptions – where parents, for example, neglect the children – should not dictate the rules for the broad majority. This very positive view of shared parenting is quite different from both law and all available guidelines in Denmark.

Regarding conflict, the report concludes that it should not rule out shared parenting, but that the focus should instead be on conflict reduction. This can be done, for example, through practical measures such as reducing the number of times the parents have to meet to hand over the children (e.g., by one parent dropping them off at daycare and the other picking them up). The report highlights the danger of considering conflict as a valid reason for avoiding shared parenting as this can give one parent an incentive for creating and maintaining conflict, effectively exposing the children to a higher level of conflict than otherwise. It is also emphasized that shared parenting may actually shield the children from the effects of conflict instead of exposing them to it. This recommendation very much stands in contrast to most Danish guidelines.

Braver and Lamb (2018) report a panel discussion on shared parenting between 12 leading international researchers. All 12 researchers agreed that children’s benefits of shared parenting could no longer be doubted and were found in areas such as: 1) lower depression, anxiety and dissatisfaction, 2) lower aggression and reduced alcohol/substance abuse, 3) better school performance and cognitive development, 4) better physical health, 5) lower smoking rates and 6) better relationships with fathers, mothers, stepparents and grandparents. They referred to literature concluding that the benefits are not due to self-selection, but that shared parenting has a causal, positive effect. The panel also addressed the question of whether shared parenting should be a legal presumption (which is currently only the case in Sweden, Belgium and four US states (Arizona, Arkansas, Kentucky and West Virginia)). In practice, this would make shared parenting the default arrangement unless concrete circumstances make it inappropriate. The experts assessed (though not unanimously) that this should be the case. It was agreed that there must be legitimate reasons for deviating from the norm, e.g., abuse/neglect, too great a distance between parents’ homes, threat of abduction and excessive gatekeeping. The majority of the experts also agreed that conflict should not prevent shared parenting, and that shared parenting does not require the parties to agree on the arrangement. The panel furthermore noted that their recommendations do not align with current practice and consider them ahead of practice. Indeed, there is once again quite a gap between these recommendations and Danish law/guidelines.

As mentioned above, shared parenting is still only rarely a legal presumption, but one such implementation has been evaluated by a range of professionals. Fabricius et al. (2018) asked four professional groups about their experiences with Arizona’s law change in 2013, including judges, attorneys, mental health staff and conciliation court staff. The Danish system is composed of largely similar groups where Agency of Family Law staff carries out similar work to conciliation court staff, including mediation between the parties and child interviews. No professional groups assessed the law negatively and most assessed it as positive overall. Specifically in relation to the children’s best interests, attorneys and mental health staff assessed it neutrally while conciliation staff and judges assessed it positively. The positive view from conciliation court staff is particularly interesting as this is the group that meets the far larger and most representative share of divorced couples.

Regarding Danish experts, researchers at the Danish Center for Social Science Research appears to have had a number of reservations regarding shared parenting around 2011 and 2012 whereas the stance appears more neutral in later publications. For example, Ottosen et al. (2011, p. 12) emphasize that the logistics of shared parenting is an additional stressor for children, that it requires that the child is robust, and that a range of other requirements need to be in place for the child to be able to handle the arrangement. An article on their website concludes from the 2012 report that equal time is not for teenagers based on the drop in prevalence for this group (but it does not mention that the satisfaction was higher for teenagers with equal time compared to those in other arrangements). Similarly, an introductory literature review in the 2011 publication takes a relatively cautious stance toward shared parenting. The report referred to the findings on contact frequency (but not duration), it referenced an article reporting that more frequent contact is bad for children if there is interparental conflict (but not evidence for the opposite position or for the view that equal time reduces conflicts), and it concluded overall that quality of contact is important whereas frequency is not (Ottosen et al., 2011, p. 26). The literature was summarized as inconclusive and when advantages of shared parenting were mentioned, potential confounds from self-selection or requirements about absence of conflict were emphasized, and it was followed by references for the quality over quantity view (Ottosen et al., 2011, p. 30). In a final summary, the report stated that for equal time to work best, it must be voluntary (not court-imposed), it requires extensive collaboration, and finally, that some results indicate that it is problematic for young children (Ottosen et al., 2011, pp. 33–34). Overall, this position aligns well with the recent position taken by Danish authorities recommending shared parenting only for the 15–20% of Danish children who are around 6–11 years old and whose parents are not in conflict but work well together.

The 2022 publication generally has a much less extensive review but presents a more neutral or positive view toward shared parenting, likely reflecting that more evidence has become available and that there is now less reason for caution. Nevertheless, it does report the finding from the 2018 publication that there was no positive effects of equal time when controlling for additional variables without mentioning the authors’ previous caution not to draw causal conclusions (Ottosen et al., 2022, p. 228). In contrast, the report presents positive effects of equal time but cautions not to draw causal conclusions (Ottosen et al., 2022, p. 236). The most positive view was possibly expressed in the 2018 publication, which highlighted research by Nielsen as well as Baude and colleagues arguing that the positive effects of shared parenting remain when taking into parent–child relationship, income and conflict (Ottosen et al., 2018, p. 247).

## 12. Concluding discussion

Over the past 60 years, the caregiving role of Danish fathers has transitioned from peripheral involvement to providing around 40% of the primary care, and at the same time spending more time with the children than mothers did one and two generations ago (see Section 2 of this article). Despite the increased role in caregiving in society in general, fathers’ share of care after divorce has lagged decades after societal development (Section 3). While there is no clear scientific consensus on all aspects, the majority of studies report benefits associated with increased father involvement up to and including equal time (Sections 5–9). Similarly, a large number of experts recommend shared parenting in the vast majority of cases (Section 11), just as the children themselves report the greatest satisfaction in shared parenting and later in adulthood assess that this is the best for children in general (Section 10). Specifically in Denmark, a substantial proportion of children report that they wish to have more time with their fathers (Section 10).

Although a causal link cannot be established, the slow transition toward shared parenting post-divorce in Denmark has coincided with law and guidelines that reflect a cautious stance toward it. Specifically, current law and guidelines are quite open to interpretation and set only a minimal framework for children’s rights to contact with both parents, yet they impose special requirements on shared parenting. The law establishes that in case of disagreement, one parent is decided to hold residency, thus effectively establishing an unequal starting point by default. In ministerial guidelines, equal parenting time as a visitation scheme has some additional relatively strict and specific requirements (regarding collaboration) that do not align with recent consensus statements and which make it difficult to establish equal time outside of mutual agreement among the parents. Guidelines from the most important Danish institution, The Agency of Family Law, have until very recently recommended against shared parenting for the vast majority of children, meaning that societal transition toward shared parenting can be said to have happened on a voluntary basis in spite of official recommendations and with a legal framework against it. Researchers at the Danish Center for Social Science Research agree that the change is largely cultural and not facilitated by law or structural changes (Ottosen et al., 2022, p. 236), and they speculate that the only documented historical decline in shared parenting – from 36 to 21% for 3-year-old children between 2013 and 2017 – was related to the authorities’ recommendations. The center itself appears to take a relative cautious stance in publications from 2011 and 2012, but a more positive stance appears present in particularly a 2018 publication and to some extent in a 2022 publication.

The most recent Agency of Family Law guidelines are less conservative, but nevertheless reference selectively the review articles that dedicate the least space to studies about young children and interparental conflict, yet express the least positive view on shared parenting. Reviews that argue that the evidence supports a positive stance on shared parenting for most families are not listed and neither are statements from leading international researchers and experts. Quantitative studies showing that children generally desire more time with their father are not mentioned either. Reference is made to studies reporting little to no impact of paternal contact frequency but not to studies reporting numerous positive effects of overall contact duration.

Taken together, current Danish institutional guidelines/law/legal practice appear to reflect a more reluctant stance on shared parenting than research evidence, children’s reports and societal practice warrants. This is not unique to Denmark but indeed appears more the rule than the exception internationally. The status is nevertheless particularly surprising given the high degree of father involvement in Danish society and Denmark’s relatively high degree of gender equality in general. With a father involvement of 40–45% in society in general, it appears in fact that the reduction to 30–35% post-divorce is a main limiting factor in achieving near-complete equality overall.

The slow implementation of research and expert opinion into Danish practice may stem in part from a principle of caution to avoid departing from traditional practice without clear evidence. It may also have been influenced by a relatively cautious stance taken by leading Danish researchers. In this context, it is worth noting that the debate presently does not focus on whether shared parenting is related to the best outcome, but whether it is *causally* related, and the main alternative explanation is that the extent of contact does not matter when taking confounding factors related to the parents into account. It may thus be argued that a departure from the stance that sole maternal residence is best for the child unless both parents agree otherwise carries primarily a risk of not having an effect. In contrast, if the effect is causal, restraint in departing from current practice restricts tens of thousands of Danish children to parenting arrangements that negatively impact their parent–child relationships, their development and their mental health, and which they themselves do not desire.

It may be mentioned in this context that divorce is not a traditional event with a traditional solution, but rather something that became common just 50–60 years ago in Denmark. The solution of maternal residence and unequal parenting time in the vast majority of cases can in itself be described as a large-scale societal experiment, which was not based on empirical evidence, and which authorities should not be afraid to revise in light of such evidence. It is particularly interesting that shared parenting appears to allow children to benefit from a good father-child relationship and good parental cooperation while the benefits of these are reduced or lost entirely in other arrangements. In contrast, skewed arrangements do not appear to offer anything unique that is not possible in shared parenting. Particularly in a society with high pre-divorce father involvement, it is worth considering whether shared parenting as a legal presumption might not be the most effective way of preventing widespread, negative divorce-related changes in parent–child relationships. An update of ministerial guidelines on visitation schemes may serve a similar function to establish equal parenting time (in the absence of official dual residence). There also appears to be some room for Agency of Family Law staff to update their guidelines and decrease the gap in parenting time within the existing rules. Of course, such changes should not exclude that the parties involved can choose another arrangement if there is agreement that it is the best, or that the authorities can rule against it in a number of cases.

## Author contributions

The author confirms being the sole contributor of this work and has approved it for publication.

## Footnotes

^1^I use the term divorce throughout the article for simplicity, but it should be noted that some studies include unmarried partners who have separated. This is also the case for the reviewed Danish studies. Given the high prevalence of children outside marriage in Denmark, I consider this a relevant inclusion.

^2^For example, for children below 1 year, fathers spent 1.7 h/day on primary caregiving and mothers spent 2.45 h/day in 2008. At age 10, fathers spent around 35 min/day while mothers spent around 55 min/day.

^3^For example, for 3-year-olds, 28% of children have “weekend contact” with an average of 5 overnights stays, and 94% reside with their mother. For these children, fathers thus provide an average of 5*0.94 + 25*0.06 = 6.2 monthly care nights, or 20.7% of the care if we treat a month as 30 days (i.e., 6.2/30 = 20.7%). Across all visitation categories for 3-year-olds, fathers provided 6% the care for 16% of the children (no contact category), 11.9% for 31% of the children (limited contact), 20.7% for 28% of the children (weekend contact), 32.4% for 17% of the children (extended contact) and 50% for 8% of the children (equal time). Adding these proportions lead to an estimate of fathers providing 19.9% of the care for 3-year-olds.
